# Prevalence of COVID-19 neurological manifestations in patients referred to the Golestan Hospital in Ahvaz between March 2020 to the March 2023

**DOI:** 10.3389/fneur.2024.1413628

**Published:** 2024-07-11

**Authors:** Alireza Mazraeh, Azarakhsh Azaran, Davood Shalilahmadi, Shahram Jalilian, Saeed Hesam

**Affiliations:** ^1^Department of Medical Virology, School of Medicine, Ahvaz Jundishapur University of Medical Sciences, Ahvaz, Iran; ^2^Department of Neurology, School of Medicine, Ahvaz Jundishapur University of Medical Sciences, Ahvaz, Iran; ^3^Department of Biostatistics and Epidemiology, School of Health, Ahvaz Jundishapur University of Medical Sciences, Ahvaz, Iran

**Keywords:** COVID-19, SARS-CoV-2, neurological manifestations, CPK, seizure, nausea, headaches, vomiting

## Abstract

Due to the abundance of ACE2 receptors in nervous system cells, the SARS-CoV-2 virus can cause damage to this system. This study aims to examine the prevalence of neurological symptoms in COVID-19 patients. In this cross-sectional observational study, 75 COVID-19 positive patients admitted to Golestan Hospital’s neurology department in Ahvaz, Iran, from March 2020 to March 2023, were investigated. Neurological clinical symptoms were categorized into three groups: central nervous system, peripheral, and muscular symptoms. The relevant information was collected from patient files, including medical history, imaging data, and laboratory test results. Statistical analysis was performed using SPSS software, employing the rank-biserial correlation coefficient (*r*), Mann–Whitney U tests, Phi correlation, Cramer’s V, and Kendall’s Tau to evaluate the prevalence and significance of neurological symptoms. The most common clinical symptoms observed were hemiparesis, dysarthria, Central Facial Palsy (CFP), ataxia, and nausea, respectively. Among these symptoms, headaches (*p* = 0.001), seizures (*p* = 0.024), and nausea (*p* = 0.046) were found to be more prevalent in younger patients. Additionally, a significant relationship was identified between the level of serum Creatine phosphokinase (CPK) and seizures (*p* = 0.024), with lower levels observed in individuals with vomiting (*p* = 0.024), and higher levels observed in individuals with CFP (*p* = 0.040). This study highlights that patients with COVID-19 may experience serious neurological symptoms. The clinical spectrum and range of neurological symptoms associated with COVID-19 were found to be diverse and extensive, emphasizing the importance of considering this infection as a potential cause of neurological disorders.

## Introduction

Coronaviruses (CoVs) are a type of enveloped positive-sense RNA viruses. They are classified into four genera: alphacoronavirus, betacoronavirus, gammacoronavirus, and deltacoronavirus (1). The COVID-19 pandemic, which is caused by the SARS-CoV-2 virus, originated in Wuhan, China, and has since spread worldwide, affecting nearly every country (2). This disease exhibits a wide range of symptoms that impact almost all physiological systems, with a particular focus on the respiratory system.

**Figure 1:**
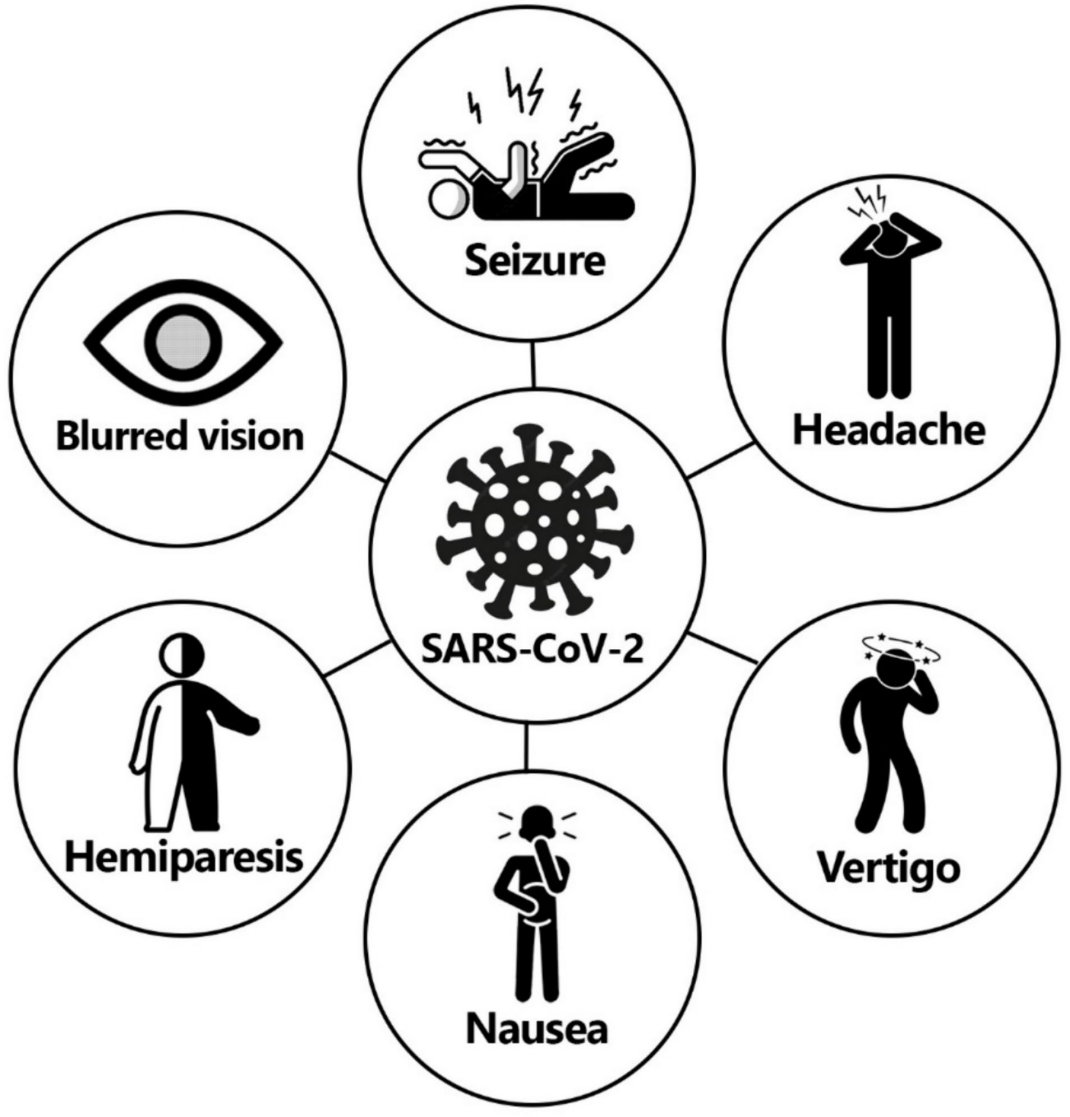
Most common neurological manifestations of COVID-19 including vertigo, blurred vision, nausea, hemiparesis, and seizure.

The expression of ACE2, a critical factor in SARS-CoV-2 infection, is significant in cells of the respiratory, renal excretory, reproductive, and digestive systems. It is also present in the cerebral cortex, brainstem, and amygdala (3). As a result, COVID-19 patients may experience various neurological symptoms (Figure 1), which can be categorized into three groups. The first group includes symptoms related to the central nervous system, such as headaches, dizziness, altered mental state, and disorientation. The second group comprises symptoms associated with the peripheral nervous system, such as decreased sense of taste and smell. The third group involves musculoskeletal symptoms. Although myalgia is a commonly reported symptom of COVID-19, there is limited literature on other musculoskeletal manifestations during the early stages of the pandemic (4, 5).

The objective of this study is to conduct a comprehensive analysis of the neurological symptoms associated with COVID-19, covering the period from March 2020 to March 2023. The aim of this research is to provide an overview of the diverse range of neurological complications observed in a cohort of hospitalized COVID-19 patients who underwent neurological evaluation. By doing so, this study seeks to enhance our understanding of the neurological symptoms induced by COVID-19, including those affecting the central nervous system, peripheral nervous system, and muscles, as well as their prevalence. Numerous studies have investigated the prevalence and types of neurological symptoms in COVID-19 patients, reporting a wide range of findings. For instance, research by Paterson et al. documented cases of encephalopathy, stroke, and peripheral neuropathies in hospitalized patients (6), while Mao et al. identified frequent occurrences of headache, dizziness, and altered mental status (7). These studies utilized various methodologies, from observational cohorts to case series, and highlighted different limitations, such as small sample sizes and lack of longitudinal follow-up. By reviewing these key studies, our research not only contextualizes its findings within a broader framework but also addresses specific gaps, such as the extended study period and comprehensive symptom analysis, thereby contributing significant new insights into the neurological impact of COVID-19. The findings of this study will offer valuable insights that can contribute to the improvement of management and rehabilitation approaches for individuals suffering from neurological disorders associated with COVID-19.

## Materials and methods

The current study is a cross-sectional descriptive observational study based on hospital and laboratory data obtained from the archived files of patients participating in the research project with code U-01045. The study was conducted with the permission of the ethics committee, and it adhered to the ethics code “IR.AJUMS.REC.1401.095.” The study population comprises the medical records of all patients admitted to the neurology department of Golestan Ahvaz Hospital during the COVID-19 pandemic, spanning from March 2020 to March 2023. Inclusion criteria for the study required patients to have tested positive for COVID-19 through Real-time PCR (RT PCR, qPCR).

In this case–control study, the researchers examined the files of patients referred to and admitted to the neurology department at Golestan Ahvaz Hospital due to neurological problems and complications affecting the central nervous system, peripheral nerves, and musculoskeletal system, all of which were caused by COVID-19. The study focused on patients who tested positive for COVID-19 through RT PCR during their hospitalization. Various clinical neurological symptoms such as headache, vertigo, decreased level of consciousness, vomiting, seizures, neuralgia, hypogeusia, ataxia, etc., were analyzed. Additionally, laboratory findings (CBC, CRP, serum Creatine phosphokinase (CPK)) and medical imaging (CT scans of the chest and brain) were extracted from the electronic and archived medical records of the patients for further analysis.

A total of 75 out of 139 hospitalized cases during the specified period were examined according to the criteria outlined in Table 1.

**Table 1:** The inclusion and exclusion criteria employed for the study were as follows.

Inclusion criteria	Exclusion criteria
Having neurological symptoms	Having a history of neurological disease prior to contracting Covid-19
Referred to the neurology department	Hospitalization in other departments of the hospital
Having a positive RT PCR test for Covid-19 during hospitalization	Not having a positive RT PCR test for Covid-19 during hospitalization
Having a brain CT scan for individuals with acute cerebrovascular disease	The absence of a brain CT scan in individuals referred with acute cerebrovascular disease
An increase in serum CPK level above 200 IU/L*	Having a CPK level less than 200 IU/L for individuals with muscle damage
Myalgia for individuals with muscle damage	The absence of myalgia in individuals with muscle damage

^*^The reference range for CPK levels is between 20 to 200 international units per liter (IU/L).

For statistical analysis of the data, SPSS version 27 software was utilized. The researchers employed various tests, including standard deviation, Mann–Whitney test, Phi correlation coefficient, Cramer’s V coefficient, agreement table test, and Kendall’s Tau. To assess the correlation between clinical symptoms and gender variables, the Cramer’s V test was employed. Furthermore, the Mann–Whitney test was utilized to compare the average age across different levels of clinical symptoms, thereby examining the relationship between age and clinical symptoms. These findings are presented in Table 2. Kendall’s Tau was employed to determine the correlation between the CRP variable and CT variable. The significance level (*p*-value) for all tests was set at 0.05. Lastly, the rank-biserial correlation coefficient (*r*) was calculated for each symptom to quantitatively measure the strength of the observed phenomenon.

**Table 2:** Characteristics and demography of patients.

Age (*n*)	Patients [male/female]	*p*-value {age/gender}	*Z (age)/**Phi (gender)
Mean	61.39 [62.13/60.62]		
Range	17–99[22–93/17–99]		
Neurological manifestations			
Headache, *n* (%)	10 (13.3) [4 (5.3)/6 (8.0)]	**0.001**/0.493	3.379/0.083
Vertigo, *n* (%)	11 (15.5) [5 (6.6)/6 (8.0)]	0.066/0.867	1.383/0.200
Nausea, *n* (%)	15 (22.4) [8 (10.6)/7 (9.3)]	**0.045**/0.720	2.009/0.440
Vomiting, *n* (%)	11 (15.5) [6 (8.0)/5 (6.6)]	0.450/0.782	0.755/0.330
Decreased level of consciousness, *n* (%)	23 (30) [13 (17.3)/10 (13.3)]	0.115/0.500	1.575/0.078
seizure, *n* (%)	10 (13.3) [4 (5.3)/6 (8.0)]	**0.024**/0.469	2.261/0.084
Acute encephalomyelitis, *n* (%)	2 (2.7) [1 (1.3)/1 (1.3)]		
Ataxia, *n* (%)	23 (30.7) [8 (10.6)/15 (20)]	0.968/0.067	0.040/0.211
CFP, *n* (%)	24 (32) [17 (22)/7 (9.3)]	0.085/**0.017**	1.722/0.277
Hemiparesis, *n* (%)	47 (62.7) [26 (34.7)/21 (28)]	0.116/0.296	1.573/0.230
Dysarthria, *n* (%)	34 (45.3) [21 (28)/12 (16)]	**0.029**/**0.046**	2.185/0.050
Blurred vision, *n* (%)	2 (2.7) [1 (1.3)/1 (1.3)]		
Hyposmia, *n* (%)	1 (1.3) [0/1 (1.3)]		
PFP, *n* (%)	1 (1.3) [0/1 (1.3)]		
DTR impairment, *n* (%)	1 (1.3) [0/1 (1.3)]		
Hypogeusia, *n* (%)	2 (2.7) [1 (1.3)/1 (1.3)]		
Quadriparesis, *n* (%)	7 (9.3) [3 (4)/4 (5.3)]	0.330/0.664	0.975/0.230
Myoclonus, *n* (%)	2 (2.7) [1 (1.3)/1 (1.3)]		
Myalgia, *n* (%)	4 (5.3) [1 (1.3)/3 (4)]	0.329/0.291	0.955/0.122

^*^The absolute value of *Z* in the Mann–Whitney test is considered significant at values above 1.961. ^**^The Phi coefficients in the Cramer’s V test indicate the relationship between variables and their value ranges between 1 and −1 the coefficient. If the value is closer to 1 or −1 the coefficient, it indicates a stronger relationship. While if it is closer to zero, it indicates a weak or no relationship. Bold values denote statistical significance at the *p* < 0.05 level.

## Results

According to the data obtained from this study, which aimed to investigate the prevalence of neurological symptoms in COVID-19 patients admitted to the neurology department of Golestan Hospital in Ahvaz, a total of 75 patients met the inclusion criteria and had no exclusion criteria.

Out of the 75 patients included in the study, 38 cases (50.7%) were male. The youngest patient was 17 years old, the oldest was 99 years old, and the mean age of the patients was 61.39 ± 17.59.

Among the patients included in the study, central nervous system involvement symptoms were observed in 10 patients (13.3%) (Table 2), with headaches in 11 patients (16.4%), and dizziness in 15 patients (22.4%), with nausea. Further information related to patients with COVID-19 with neurological manifestations is given in Table 2.

A review of laboratory criteria and clinical symptoms showed that approximately 21% (16 patients) had leukocytosis and 53% (39 patients) had lymphopenia. Neutrophilia was observed in 77% (58 patients), and neutropenia in 2.6% (2 patients) of the patients studied. Of the patients tested for CPK, 63% (39 patients) had values above the normal range. 53% (39 patients) had a lower-than-normal red blood cell count, and 2.6% (2 patients) had a higher amount than the normal red blood cell count. Platelet counts were lower than normal in 6% (5 patients) and higher than normal in about 10% (8 patients) of patients. There was no significant relationship between the differential white blood cell count, including lymphocyte-to-neutrophil ratio and total white blood cell count, and clinical symptoms. Although neutrophilia was clearly observed in patients, there was no significant correlation with neurological clinical symptoms. There was also no significant correlation between the red blood cell count and clinical symptoms. The platelet count was not significantly related to clinical symptoms either.

Among the inflammatory factors, qualitative CRP and quantitative CPK tests were performed for the patients. A significant correlation was observed between a lower incidence of vomiting and CPK levels (*Z* = −2.252, *p* = 0.024). The occurrence of seizures was also significantly related to CPK levels (*Z* = −2.263, *p* = 0.024). In addition, CPK levels were significantly increased in individuals with myalgia (*Z* = 2.593, *p* = 0.010). There was no significant correlation between CRP levels and clinical symptoms, but CRP levels had a significant correlation with the degree of lung involvement seen on chest CT scan (*p* = 0.004).

In order to enhance the study’s reliability and obtain a more comprehensive understanding of the probability of Type II errors, a power analysis was conducted. The rank-biserial correlation coefficient (*r*) was used as the effect size measure, which is appropriate for the non-parametric Mann–Whitney U tests employed in this study.

Regarding headaches, the analysis revealed an effect size of *r* = 0.39, which is considered to be of medium effect. With this effect size and the sample size of the study, the power to detect this effect is approximately 78%. For seizures, the calculated effect size was *r* = 0.26, indicating a small to medium effect. The power to detect this effect with the given sample size is about 52%. In the case of nausea, the effect size was calculated as *r* = 0.23, also indicating a small to medium effect. The power to detect this effect is approximately 45%.

These findings indicate that the study has moderate power for detecting the effect on headaches, but lower power for seizures and nausea. This suggests a higher risk of Type II errors for the latter two symptoms.

## Discussion

Amidst a growing body of literature highlighting the neurological sequelae of COVID-19, our study presents a unique contribution by focusing on the prevalence of neurological symptoms specifically among COVID-19 patients admitted to our neurology department. While existing research provides valuable insights into the broad spectrum of neurological impairments associated with the virus, our study offers a specialized perspective, emphasizing the manifestations observed within a clinical neurology setting. By examining a cohort of patients receiving care within this specialized context, we provide nuanced insights into the frequency and nature of neurological symptoms in a subset of COVID-19 cases, thereby enhancing our understanding of the virus’s impact on the nervous system in a clinical setting. This focused approach not only complements existing research but also facilitates targeted clinical interventions and management strategies tailored to the unique needs of COVID-19 patients presenting with neurological manifestations.

In a study by Romero-Sánchez et al. (8) in 2020, which included 841 hospitalized patients in Spain, symptoms such as myalgia (17.2%), headache (14.1%), and dizziness (6.1%) were more common in the early stages of infection. Olfactory (4.9%) and gustatory (6.2%) disturbances were less frequent and milder, while neuromuscular abnormalities (3.1%), cerebrovascular disease (1.7%), seizures (0.7%), movement disorders (0.7%), encephalopathy (one case), GBS (one case), and visual neuritis (one case) were also reported. Neurological complications were the main cause of death in 4.1% of all study participants. The study suggests that some neurological symptoms, such as myalgia, may decrease after the onset of neurological symptoms, and it was not possible to obtain a history of myalgia from people who were unconscious during admission to the emergency department. Additionally, patients were not routinely assessed for smell and taste in A&E departments, resulting in missing data in this area. Based on the present study in the neurology department, the occurrence of symptoms related to the nervous system is naturally higher, while general symptoms are less prevalent, compared to a study with a random sample regardless of neurological focus.

In a study by Elodie Meppiel et al. (9) in 2021, which included 222 COVID-19 patients with neurological symptoms from 46 centers in France, the most frequent neurological diseases associated with COVID-19 were encephalopathy (30.2%), acute ischemic cerebrovascular syndrome (25.7%), encephalitis (9.5%), and GBS (6.8%). CSF was analyzed in 97 patients (43.7%), and 18 patients (18.6%) had pleocytosis, while two patients with encephalitis had positive PCR results for COVID-19. Although many cases of encephalopathy were observed during the study, these patients were not included in the study due to exclusion criteria. In a comprehensive study by Cervantes et al. (10) in 2022, conducted in 179 hospitals in 24 countries, out of 16,225 patients who were available for registry with hospital discharge status, 2,092 individuals (12.9%) had serious neurological symptoms, including 1,656 patients (10.2%) with encephalopathy during hospitalization, 331 patients (2.0%) with stroke, 243 patients (1.5%) with seizures, and 73 patients (0.5%) with meningitis/encephalopathy during hospitalization or at the time of discharge. Excluding patients with encephalopathy who were excluded due to neurological exclusion criteria, and focusing on the study population (neurology patients), given that stroke symptoms are typically among the specific symptoms of neurology, the prevalence of neurological symptoms in this study is similar to that of the present study.

In a study conducted by Shen et al. (11) in Shanghai, China in 2023 on patients hospitalized with mild to moderate COVID-19 and diagnosed with the Omicron strain, 169 out of 351 patients (48.1%) presented with neurological symptoms, the most common being fatigue/weakness (25.1%) and muscle pain (20.7%), while cerebrovascular disease (0.9%), impaired consciousness (0.6%), and seizures (0.6%) were rare. Younger age (*p* = 0.001) and female gender (*p* = 0.026) were associated with a higher proportion of neurological symptoms. Patients with neurological symptoms had lower creatine kinase levels, with fatigue/weakness (25.1%) being the most common neurological sign. Other CNS symptoms (38.5%) included headache (13.7%), dizziness (13.4%), emotional disturbance (4.9%), cerebrovascular disease (0.9%), impaired consciousness (0.6%), and seizures (0.6%), while 105 patients (29.9%) had PNS symptoms such as muscle pain (20.7%), taste disturbance (5.7%), smell disturbance (6.3%), visual disturbance (5.4%), and neuralgia (0.4%). The results of this study differed significantly from a previous study, where significant differences in dizziness and Central Facial Palsy (CFP) symptoms were found in favor of males (*p* = 0.017 and *p* = 0.046, respectively). However, like the previous study, the neurological complications that had a significant correlation with age were more common in younger ages (headache, seizures, nausea). The prevalence of seizure symptoms and other neurological symptoms in this study differed significantly from the Shanghai study. The reason for this difference may be due to differences in the description of symptoms, genetic factors in the study populations, or geographic and regional differences in underlying diseases. It should also be noted that the Shanghai study was conducted on individuals infected with the Omicron variant, while the current study did not differentiate between COVID-19 variants. Further research and studies are needed to identify the main cause of these differences.

The power analysis conducted using the rank-biserial correlation coefficient (*r*) has demonstrated that our study exhibits satisfactory power (78%) to detect a medium effect (*r* = 0.39) in relation to headaches. However, in the case of seizures and nausea, the power is comparatively lower (52 and 45% respectively), thereby indicating an elevated risk of Type II errors for these symptoms. The effect sizes for all three symptoms range from small to medium, with headaches exhibiting the most pronounced effect. These findings emphasize the importance of exercising caution when interpreting the results, particularly with regards to seizures and nausea, as the diminished power heightens the likelihood of failing to identify true effects. Future studies incorporating larger sample sizes would be advantageous in enhancing the power to consistently detect these more subtle effects.

In a study of 62 adult COVID-19 patients in Wuhan, China by Xiao et al. (12), laboratory results showed leucopenia (31%) and lymphopenia (42%) which is consistent with the current study. Two studies published in Frontiers in Immunology highlight that elevated CRP levels are strongly associated with severe forms of COVID-19. These findings indicate that high CRP levels are linked to increased mortality, the requirement for ventilators, and admissions to the intensive care unit (ICU). Specifically, CRP values above 100 mg/L are found to significantly correlate with unfavorable outcomes, including higher mortality rates and the progression of severe disease, which also has a relatively high consistency with the current study (13, 14).

Marco Meglio et al. conducted a study to examine the role of elevated CPK levels in encephalopathic COVID-19 patients. The study revealed that although many patients exhibited elevated CPK levels, these levels were not found to be a significant prognostic factor for the development of post-acute sequelae of COVID-19. Furthermore, the study’s findings emphasized that increased CPK levels were associated with severe COVID-19 cases and higher mortality rates, particularly among male and diabetic patients (15). However, a separate study by Friedman et al., published in the Neurohospitalist, demonstrated that elevated CPK levels did not consistently correlate with poorer outcomes. Instead, myalgia and mild disease were often observed in conjunction with higher CPK levels, suggesting that elevated CPK levels may not always indicate a severe disease or a higher risk of mortality (16). These studies offer various perspectives on the importance of CPK levels in COVID-19, thereby substantiating the intricate correlation between CPK elevation and the severity of COVID-19.

Based on the results of this study and similar studies, and considering the relatively short intervals between viral outbreaks such as SARS and MERS that have occurred in recent decades, and the possibility of new outbreaks in the near future, with the occurrence of multiple symptoms including involvement of various systems in the nervous system, it is suggested that COVID-19 and the coronavirus family, in general, be considered as a possible cause of disease, along with other possible contributing factors, during the diagnosis and treatment of neurological diseases. Accordingly, diagnostic and therapeutic strategies, including necessary evaluations such as laboratory measurement of inflammatory factors, should be adopted.

It is recommended that careful evaluation of neurological symptoms be conducted during the history taking process in emergency and neurology departments. Additionally, the assessment should include respiratory symptoms and the involvement of the olfactory and gustatory systems, excluding cases with impaired consciousness or dysarthria. This is imperative, as patients or their companions may overlook these symptoms.

The study emphasizes the significant neurological impact of COVID-19 on patients, emphasizing the wide range and extensive nature of symptoms that encompass central nervous system complications such as headaches, dizziness, altered mental state, and disorientation, as well as peripheral symptoms such as decreased sense of taste and smell, and musculoskeletal issues. Recognizing these symptoms is imperative for enhancing patient management and treatment strategies. By comprehending the prevalence and characteristics of these neurological manifestations, healthcare providers can improve diagnostic accuracy, customize rehabilitation approaches, and implement more effective management protocols for patients with COVID-19-induced neurological disorders. This comprehensive analysis not only contributes to the broader comprehension of COVID-19’s neurological effects but also facilitates targeted clinical interventions, ensuring improved patient outcomes and mitigating long-term complications.

Clinical implications and specific recommendations for healthcare practitioners can enhance the management of COVID-19-related neurological complications. These recommendations include enhanced screening, early interventions, regular monitoring, education, policy updates, and support for research. By implementing these measures, healthcare practitioners can improve patient outcomes and the overall quality of care.

Despite the valuable insights gleaned from our study, it is important to acknowledge several limitations that may affect the interpretation and generalizability of our findings.

Potential biases in data collection can arise from several factors, including single-center study design, documentation variability, sample size limitations, patient selection bias, and temporal factors. The single-center nature of our study restricts the extrapolation of our results to broader populations, highlighting the need for multi-center studies to validate our findings across diverse settings. Additionally, the retrospective design of our study introduces inherent biases and limitations associated with data collection and documentation practices. Variability in reporting and documentation of neurological symptoms among healthcare providers may have led to underestimation or misclassification of certain symptoms, potentially influencing the observed prevalence rates. Moreover, the relatively modest sample size of our cohort may limit the statistical power to detect associations or trends, warranting cautious interpretation of the results. While the present study provides valuable information on the neurological manifestations of COVID-19, the small sample size requires cautious interpretation of the results. Comparisons with larger studies and future research with larger and more diverse groups are necessary to confirm and extend these findings. This approach will ultimately increase our understanding of the neurological effects of COVID-19 and improve patient care. Future prospective studies with larger, more diverse cohorts are warranted to address these limitations and provide further insights into the neurological manifestations of COVID-19.

## Conclusion

To enhance the management of COVID-19 patients with neurological symptoms, it is recommended that clinical practice guidelines be updated to include a comprehensive neurological assessment for all patients. This should involve regular screening for common neurological symptoms such as headaches, dizziness, and changes in mental state, as well as peripheral manifestations such as loss of smell and muscle pain. Additionally, healthcare facilities should have the necessary resources to conduct thorough diagnostic tests, including brain imaging and measurement of serum CPK levels, in order to promptly identify and address acute cerebrovascular diseases and neuromuscular complications.

From a policy perspective, it is essential to ensure that all healthcare providers receive training on the neurological implications of COVID-19, enabling them to effectively recognize and manage these symptoms. Policymakers should also consider incorporating these guidelines into national health protocols to standardize care and improve outcomes for patients with COVID-19-related neurological issues. Furthermore, support for continued research and data collection on the neurological effects of COVID-19 is crucial in order to refine treatment approaches and develop targeted interventions.

By implementing these recommendations, healthcare systems can enhance diagnostic accuracy, tailor treatments more effectively, and ultimately improve patient care for individuals affected by the neurological impacts of COVID-19.

## Data availability statement

The original contributions presented in the study are included in the article/supplementary material, further inquiries can be directed to the corresponding author.

## Ethics statement

The studies involving humans were approved by the Research Ethics Committees of Ahvaz Jundishapur University of Medical Sciences, Ahvaz, Iran (Research project code: U-01045/Approval ID: IR.AJUMS.REC.1401.095). The studies were conducted in accordance with the local legislation and institutional requirements. Written informed consent for participation was not required from the participants or the participants' legal guardians/next of kin in accordance with the national legislation and institutional requirements.

## Author contributions

AM: Writing – original draft, Methodology, Investigation. AA: Writing – original draft, Visualization. DS: Writing – original draft, Methodology, Data curation. SJ: Writing – review & editing, Writing – original draft, Supervision, Project administration, Methodology, Conceptualization. SH: Writing – original draft, Formal analysis.

## References

[1] Acharya A Kevadiya BD Gendelman HE Byrareddy SN. SARS-CoV-2 infection leads to neurological dysfunction. J Neuroimmune Pharmacol. (2020) 15:167–73. doi: 10.1007/s11481-020-09924-9, PMID: 32447746 PMC7244399

[2] Mehta SK Sunder A. Getting paralysed after COVID: Guillain–Barre syndrome. J Family Med Prim Care. (2021) 10:2706–8. doi: 10.4103/jfmpc.jfmpc_2454_20, PMID: 34568159 PMC8415676

[3] Lukiw WJ Pogue A Hill JM. SARS-CoV-2 infectivity and neurological targets in the brain. Cell Mol Neurobiol. (2022) 42:217–24. doi: 10.1007/s10571-020-00947-7, PMID: 32840758 PMC7445393

[4] Payus AO Lin CLS Noh MM Jeffree MS Ali RA. SARS-CoV-2 infection of the nervous system: a review of the literature on neurological involvement in novel coronavirus disease-(COVID-19). Bosn J Basic Med Sci. (2020) 20:283. doi: 10.17305/bjbms.2020.4860 32530389 PMC7416180

[5] Ramani SL Samet J Franz CK Hsieh C Nguyen CV Horbinski C. Musculoskeletal involvement of COVID-19: review of imaging. Skeletal Radiol. (2021) 50:1763–73. doi: 10.1007/s00256-021-03734-7, PMID: 33598718 PMC7889306

[6] Paterson RW Brown RL Benjamin L Nortley R Wiethoff S Bharucha T. The emerging spectrum of COVID-19 neurology: clinical, radiological and laboratory findings. Brain. (2020) 143:3104–20. doi: 10.1093/brain/awaa240, PMID: 32637987 PMC7454352

[7] Mao L Jin H Wang M Hu Y Chen S He Q. Neurologic manifestations of hospitalized patients with coronavirus disease 2019 in Wuhan, China. JAMA Neurol. (2020) 77:683–90. doi: 10.1001/jamaneurol.2020.1127 32275288 PMC7149362

[8] Romero-Sánchez CM Díaz-Maroto I Fernández-Díaz E Sánchez-Larsen Á Layos-Romero A García-García J. Neurologic manifestations in hospitalized patients with COVID-19. The ALBACOVID Registry. (2020) 95:e1060–70. doi: 10.1212/WNL.0000000000009937 PMC7668545 32482845

[9] Meppiel E Peiffer-Smadja N Maury A Bekri I Delorme C Desestret V. Neurologic manifestations associated with COVID-19: a multicentre registry. Clin Microbiol Infect. (2021) 27:458–66. doi: 10.1016/j.cmi.2020.11.005, PMID: 33189873 PMC7661948

[10] Cervantes-Arslanian AM Venkata C Anand P Burns JD Ong CJ LeMahieu AM. Neurologic manifestations of severe acute respiratory syndrome coronavirus 2 infection in hospitalized patients during the first year of the COVID-19 pandemic. Crit Care Explorat. (2022) 4:e0686. doi: 10.1097/CCE.0000000000000686, PMID: 35492258 PMC9042584

[11] Shen X Wang P Shen J Jiang Y Wu L Nie X. Neurological manifestations of hospitalized patients with mild to moderate infection with SARS-CoV-2 omicron variant in Shanghai, China. J Infect Public Health. (2023) 16:155–62. doi: 10.1016/j.jiph.2022.12.005, PMID: 36535135 PMC9726211

[12] Xu X-W Wu X-X Jiang X-G Xu K-J Ying L-J Ma C-L. Clinical findings in a group of patients infected with the 2019 novel coronavirus (SARS-Cov-2) outside of Wuhan, China: retrospective case series. BMJ. (2020):368. doi: 10.1136/bmj.m606 PMC7224340 32075786

[13] Paranga TG Pavel-Tanasa M Constantinescu D Plesca CE Petrovici C Miftode IL. Comparison of C-reactive protein with distinct hyperinflammatory biomarkers in association with COVID-19 severity, mortality and SARS-CoV-2 variants. Front Immunol. (2023) 14:1213246. doi: 10.3389/fimmu.2023.1213246, PMID: 37388734 PMC10302717

[14] Luan YY Yin CH Yao YM. Update advances on C-reactive protein in COVID-19 and other viral infections. Front Immunol. (2021) 12:720363. doi: 10.3389/fimmu.2021.720363, PMID: 34447386 PMC8382792

[15] Meglio M. Creatine kinase levels not considered risk factor for COVID-19 post-acute sequelae. Neurol Live. (2023)

[16] Friedman SA Charmchi Z Silver M Jacoby N Perk J Anziska Y. Skeletal muscle manifestations and creatine kinase in COVID-19. Neurohospitalist. (2022) 12:597–606. doi: 10.1177/19418744221105961, PMID: 36147765 PMC9160579

